# Sickle cell trait is not associated with chronic kidney disease in adult Congolese patients: a clinic-based, cross-sectional study

**DOI:** 10.5830/CVJA-2014-076

**Published:** 2015

**Authors:** K Mukendi, FB Lepira, KE Sumaili, MN Nseka, PK Kayembe

**Affiliations:** Division of Nephrology and Hypertension, Department of Internal Medicine, University of Kinshasa Hospital, Kinshasa, Democratic Republic of Congo; Division of Nephrology and Hypertension, Department of Internal Medicine, University of Kinshasa Hospital, Kinshasa, Democratic Republic of Congo; Division of Nephrology and Hypertension, Department of Internal Medicine, University of Kinshasa Hospital, Kinshasa, Democratic Republic of Congo; Division of Nephrology and Hypertension, Department of Internal Medicine, University of Kinshasa Hospital, Kinshasa, Democratic Republic of Congo; School of Public Health/University of Kinshasa, Kinshasa, Democratic Republic of Congo

**Keywords:** chronic kidney disease, determinants, sickle cell trait, black Africans

## Abstract

**Objective:**

The aim of this study was to evaluate the determinants of chronic kidney disease (CKD) with special emphasis on sickle cell trait (SCT).

**Methods:**

Three hundred and fifty-nine patients (171 men and 188 women), aged 18 years or older, with reduced kidney function (eGFR < 90 ml/min/1.73 m^2^) and seen at secondary and tertiary healthcare in Kinshasa were consecutively recruited in this cross-sectional study. Serum creatinine and haemoglobin electrophoresis were performed in each patient. CKD was defined as < 60 ml/min/1.73 m^2^. Logistic regression analysis was used to assess determinants of CKD with a special emphasis on SCT. A *p*-value < 0.05 defined the level of statistical significance.

**Results:**

SCT was present in 19% of the study population; its frequency was 21 and 18% (*p* > 0.05) in patients with and without CKD, respectively. In multivariate analysis, sickle cell trait was not significantly (OR: 0.38; 95% CI: 0.559–1.839; *p* = 0.235) associated with CKD; the main determinants were dipstick proteinuria (OR: 1.86; 95% CI: 1.094–3.168; *p* = 0.02), the metabolic syndrome (OR: 1.69; 95% CI: 1.033–2.965; *p* = 0.03), haemoblobin ≥ 12 g/dl (OR: 0.36; 95% CI: 0.210–0.625; *p* = 0.001), and personal history of hypertension (OR: 2.16; 95% CI: 1.202–3.892; *p* = 0.01) and of diabetes mellitus (OR: 2.35; 95% CI: 1.150–4.454; *p* = 0.001).

**Conclusion:**

SCT was not an independent determinant of CKD in the present case series. Traditional risk factors emerged as the main determinants of CKD.

## Abstract

Chronic kidney disease (CKD) and end-stage renal disease (ESRD) are associated with significant cardiovascular (CV) and renal morbidity and mortality rates, with substantial economic burden.^1^,^2^ Therefore, early identification of CKD patients at high risk of progression is urgently needed for early and targeted treatment to improve patient care.^1^-^3^ Diabetes and hypertension are the primary risk factors for CKD and ESRD but do not fully account for CKD and ESRD risk.^1^-^3^ Marked variability in the incidence of CKD suggests that factors other than diabetes and hypertension contribute to its aetiology.^4^

Family studies have suggested a genetic component to the aetiology of CKD and ESRD.^5^ In African Americans, high-risk common variants in the Apol1/MYH9 locus may explain up to 70% of the differences in ESRD rates between European and African Americans.^5^ While this finding has great implications for ESRD, the identification of additional risk factors for CKD, including genetic loci in association with estimated glomerular filtration rate (eGFR), may help to advance our understanding of the underpinnings of CKD in African Americans.^5^ In this era of identifying genetic risk factors for kidney disease, it may be appropriate to revisit one of the most common genetic disorders: sickle cell haemoglobinopathies.^5^

In this regard, sickle cell trait (SCT), present in approximately 7–9% of African Americans, has been reported to be a potential candidate gene.^6^ However, conflicting reports exist as to whether SCT is a risk factor for the progression of nephropathy.^6^,^7^ Haemoglobin S (HbS) was selected for in Africa because of the protection it affords from malarial infection, a scenario similar to the protection from trypanosomal infection provided by heterozygosity for APOL1 nephropathy risk variants.^6^

Whereas APOL1 contributes to risk for nephropathy in an autosomal recessive inheritance pattern, HbS reportedly had a dominant effect on risk, with SCT being associated with ESRD.^6^ In line with this finding, a few small studies on African Americans reported HbS as an independent risk factor for CKD and ESRD.^8^ However, other studies using a large sample of African Americans stated that SCT was not independently associated with susceptibility to ESRD in African Americans,^6^ highlighting the need for further studies in other populations such as those of sub-Saharan Africa where SCT is prevalent.

Although SCT is very prevalent in black Africans,^9^ few studies have been conducted to assess the association between SCT and CKD.^10^ In Democratic Republic of Congo (DRC), the prevalence of CKD and SCT has been reported to be 12% and 17–24%, respectively.^11^-^13^ No study has evaluated the frequency of SCT among CKD patients to assess its association with reduced kidney function. Therefore, the aim of this clinic-based, cross-sectional study was to assess the potential association between SCT and CKD among adult Congolese patients.

## Methods

From 30 April to 24 August 2012, all consecutively appearing patients with known CKD seen in tertiary care (University of Kinshasa Hospital) and those with diabetes or hypertension regularly followed in secondary care (General Hospital of Kinshasa and Saint Joseph Hospital) were asked to participate in this cross-sectional study. Inclusion criteria were: age ≥ 18 years, antihypertensive treatment for at least three months, and written informed consent.

The sample was a convenient one. Self-reported alcohol use, smoking habits, personal and family history of hypertension or diabetes, family history of sickle cell anaemia (SCA) and measure of adiposity [body mass index (BMI) and waist circumference (WC)] were obtained for all patients. Excessive alcohol intake was defined as regular intake of two or more glasses per day of beer or equivalent for at least one year, knowing that one glass of beer contains 10 g of alcohol.^14^ Smoking was defined as regular consumption of at least one cigarette per day for more than five years or having stopped smoking for less than five years.^15^ Overweight and obesity were defined as BMI ≥ 25 and ≥ 30 kg/m^2^, respectively.^16^ Central obesity was defined as WC > 94 cm in men > 80 cm in women.^17^

Seated blood pressure (BP) was measured using an electronic device Omron M3 on the left arm at the level of the heart after five minutes’ rest. Three consecutive BP measurements at two-minute intervals were made and the mean of the last two readings was used for analysis. Pulse pressure (PP) was calculated as systolic blood pressure (SBP) minus diastolic blood pressure (DBP) and was considered increased when > 60 mmHg.^18^ Hypertension was defined as BP ≥ 140/90 mmHg or current use of antihypertensive, whatever the level of BP.^18^ Heart rate was counted for a full minute.

A 12-hour overnight fasting blood sample was collected from each patient for measurement of haemoglobin (Hb), total cholesterol (TC) and its sub-fractions [low-density lipoprotein cholesterol (LDL-C), high-density lipoprotein cholesterol (HDL-C)], triglycerides (TG), glucose, uric acid and creatinine levels at the Laboratory of the National AIDS Control Program (NACP). LDL-C was calculated using the Friedewald formula.^19^ The metabolic syndrome (MetS) was defined according to 2009 consensus criteria.^17^ Diabetes was defined as plasma glucose > 7 mmol/l or current use of antidiabetic drugs, whatever the level of blood glucose.^20^ A uric acid level > 416 μmol/l was defined as hyperuricaemia.^21^

Serum creatinine concentrations were analysed based on a modified Jaffe reaction (picric acid) using an automated device (Dimension® XPand® Plus, Siemens). Estimated glomerular filtration rate (eGFR) was calculated using the modification of diet in renal disease (MDRD) equation,^22^ based on serum creatinine levels calibrated as described elsewhere.^23^ The Combur 9 test (Roche, France) was used on morning spot urine collections to determine semi-quantitative proteinuria; positive proteinuria was defined as Combur 9 test ≥ 1+.^24^ According to KDOQI,^25^ reduced kidney function and CKD were defined as GFR < 90 ml/min/1.73 m^2^ and < 60 ml/min/1.73 m^2^, respectively.

Haemoglobin types were determined using isoelectrofocalisation electrophoresis (Capillaris, France) at the laboratory of Monkole Hospital in Kinshasa. This analytical method results in elution of haemoglobin variants and determines the proportion of these variants relative to the total haemoglobin concentration.^26^ It has been shown to be a reliable determinant of the HbS concentration and allows for the determination of HbS and HbC traits.^26^

## Statistical analysis

Data are expressed as mean ± standard deviation (SD) or relative frequency in percentages. Chi-square and Student’s *t*-tests were used for comparing categorical and normally distributed continuous variables, respectively. The Mann–Whitney test was used for non-normally distributed continuous variables. Multiple logistic regression analysis and the likelihood ratio method were performed with CKD as the dependent variable for the assessment of the strength and independence of association with CKD risk factors, among them, SCT alone or in interaction with hypertension or diabetes. Adjusted odds ratio (aOR) and their 95% confidence intervals (CI) were calculated for each variable. All statistical analyses were performed with SPSS for Windows, version 12.0 at the Division of Epidemiology and Biostatistics of Kinshasa Public Health School, University of Kinshasa.

## Results

A total of 359 patients with reduced kidney function (198 women and 161 men) were recruited in this study. Clinical characteristics of the study population as a whole and by renal functional status are given in Table 1. Their mean age was 56 ± 15 years; they had on average a BMI of 26 ± 5 kg/m^2^, WC of 90 ± 14 cm, SBP of 143 ± 26 mmHg and DBP of 83 ± 13 mmHg. A family history of sickle cell disease (FH-SCD) was present in 6% of patients. Average levels of TC, HDL-C, TG, glucose, uric acid, Hb and eGFR were 5.32 ± 2.22 mmol/l, 1.49 ± 0.59 mmol/l, 1.31 ± 0.65 mmol/l, 8.16 ± 4.94 mmol/l, 360 ± 159 mmol/l, 11 ± 2.40 g/dl and 59 ± 46 ml/min/1.73 m2, respectively (Table 2).

**Table 1:** Clinical characteristics of the study population as a whole and by renal functional status

*Variable*	*n*	*Whole group (n = 298)*	*CKD– (n =171)*	*CKD+ (n = 188)*	*p-value*
Age (years)	359	56 ± 15	64 ± 10	64 ± 10	
Gender (%)	359				
Males		45	41	48	0.548
Females		55	59	52	
FH-SCD (%)	359	6	7	6	0.897
BMI (kg/m^2^)	359	25 ± 5	25 ± 5	26 ± 6	0.341
WC (cm)	359	90 ± 14	88 ± 12	92 ± 16	0.009
SBP (mmHg)	359	143 ± 26	136 ± 24	151 ± 26	0.001
DBP (mmHg)	359	83 ± 13	81 ± 14	85 ± 15	0.001
PP (mmHg)	359	60 ± 20	54 ± 19	66 ± 21	0.001
Causes of CKD	359				
HT (%)		39	43	35	0.248
DM (%)		44	37	51	0.010
GN (%)		14	15	13	0.693
Other (%)		3	5	1	0.072
Stages of CKD	359				
Stage 3 (%)				40	
Stage 4 (%)				38	
Stage 5 (%)				21	

Data are expressed as mean ± standard deviation (SD) or relative frequency (%). FH-SCD, family history of sickle cell disease; BMI, body mass index; WC, waist circumference; SBP, systolic blood pressure; DBP, diastolic blood pressure; PP, pulse pressure; HT, hypertension; DM, diabetes mellitus; GN, glomerulonephritis; CKD, chronic kidney disease.

**Table 2:** Biological characteristics of the study population as a whole and by renal functional status

*Variable*	*n*	*Whole group (n = 359)*	*CKD– (n = 171)*	*CKD+ (n = 188)*	*p-value*
Hb (g/dl)	330	11 ± 2.40	12 ± 2.10	10 ± 2.20	0.001
Blood glucose (mg/dl)	350	8.16 ± 4.94	8.94 ± 4.61	7.50 ± 5.16	0.005
TC (mmol/l)	294	5.32 ± 2.22	5.14 ± 1.73	5.55 ± 2.22	0.312
LDL-C (mmol/l)	294	3.04 ±1.65	2.99 ± 1.60	3.07 ± 1.70	0.662
HDL-C (mmol/l)	294	1.49 ± 0.59	1.58 ± 0.46	1.39 ± 0.67	0.014
TG (mmol/l)	294	1.31 ± 0.65	1.22 ± 0.54	1.42 ± 0.75	0.017
Uric acid (mmol/l)	313	360 ± 159	277 ± 100	442 ± 165	0.001
Creatinine (μmol/l)	359	87 ± 44	80 ± 24	94 ± 54	0.001
eGFR (ml/min/1.73 m^2^)	359	59 ± 46	95 ± 39	26 ± 20	0.001
Dipstick proteinuria (%)	359	24	24	37	0.001

Data are expressed as mean ± standard deviation (SD) or relative frequency (%). Hb, haemoglobin; TC, total cholesterol; LDL-C, low-density lipoprotein cholesterol; HDL-C, high-density lipoprotein cholesterol; TG, triglycerides; eGFR, estimated glomerular filtration; CKD, chronic kidney disease.

CKD was present in 188 patients (52%), of whom 40, 38 and 21% had CKD stage 3, 4 and 5, respectively (Tables 1, 2). The main causes of CKD were diabetes (44%), hypertension (39%), glomerulonephritis (14%) and other conditions (3%). Family history of sickle cell disease was present in 7 and 6% of patients with and without CKD, respectively; the difference was not statistically significant (*p* > 0.05). Compared to patients without CKD, those with CKD had on average higher levels of WC (92 ± 16 vs 88 ± 12 cm; *p* = 0.009), SBP (151 ± 26 vs 136 ± 24; *p* = 0.001), DBP (85 ± 15 vs 81 ± 13 mmHg; *p* = 0.001) and PP (66 ± 21 vs 54 ± 19 mmHg; *p* = 0.001). They also had higher levels of TG (1.42 ± 0.75 vs 1.22 ± 0.54 mmol/l; *p* = 0.017) and uric acid (442 ± 165 vs 277 ± 100 mmol/l; *p* = 0.001), and lower levels of HDL-C (1.39 ± 0.67 vs 1.58 ± 0.46 mmol/l; *p* = 0.014), glucose (7.5 ± 5.16 vs 8.94 ± 4.61) and Hb (10 ± 2.20 vs 12 ± 2.10 g/dl; *p* = 0.001). The proportion of subjects with proteinuria was also higher in CKD patients (37 vs 24%; *p* = 0.001).

Table 3 summarises the distribution of CKD risk factors in the study population as a whole and by renal functional status. SCT was present in 19% of patients in the entire group, and 23 and 18% of those with and without CKD, respectively; the observed difference did not reach the level of statistical significance. Patients with CKD also had higher rates of the MetS (31 vs 24%; *p* = 0.001), anaemia (72 vs 42%; *p* = 0.001) and elevated PP (60 vs 39%, *p* = 0.001). Clinical and biological characteristics of CKD patients by Hb status are depicted in Table 4. Compared to CKD patients with normal Hb levels, those with SCT showed on average higher uric acid levels (560 ± 159 vs 413 ± 153 mmol/l; *p* = 0.001) and lower Hb levels (9 ± 1.80 vs 10 ± 2.20 g/dl; *p* = 0.001).

**Table 3:** CKD risk factors among the study population as a whole and by renal functional status

*Variable*	*n*	*Whole group (n = 359)*	*CKD– (n = 171)*	*CKD+ (n = 188)*	*p-value*
HbAS (%)	330	19	18	21	0.715
Smoking (%)	359	4	5	3	0.499
Alcohol (%)	359	4	4	4	0.763
Overweigh/obesity (%)	359	29	29	30	0.715
MetS (%)	359	27	24	31	0.007
Anaemia (%)	330	27	42	72	0.001
Elevated PP (%)	359	49	39	60	0.001

Data are expressed as mean ± standard deviation (SD) or relative frequency (%). HbAS, sickle cell trait; MetS, the metabolic syndrome; PP, pulse pressure.

**Table 4:** Clinical and biological characteristics of CKD patients by haemoglobin genotype status

*Variable*	**n**	*HbAA (n =149)*	*HbAS (n = 39)*	*p-value*
Age, years	188	56 ± 15	55 ± 17	0.808
Gender (%)	188			
Males		44	67	0.017
Females		56	33	
BMI (kg/m2)	188	25 ± 5	26 ± 6	0.189
WC (cm)	188	88 ± 12	89 ± 13	0513
SBP (mmHg)	188	150 ± 21	158 ± 29	0.063
DBP (mmHg)	188	85 ± 13	86 ± 16	0.550
PP mm Hg	188	64 ± 20	71 ± 21	0.061
Hb (g/dl)	177	10 ± 2.20	09 ± 1.80	0.001
Glucose (mmol/l)	181	7.39 ± 4.00	7.83 ± 2.66	0.613
Uric acid (mmol/l)	157	413 ± 153	560 ± 159	0.001
TC (mmol/l)	133	5.55 ± 2.29	5.68 ± 2.32	0.570
LDL-C (mmol/l)	133	3.07 ± 1.70	3.12 ± 1.75	0.467
HDL-C (mmol/l)	133	1.36 ± 0.72	1.49 ± 0.43	0.398
TG (mmol/l)	133	1.38 ± 0.75	1.52 ± 0.79	0.397
Creatinine (μmol/l)	188	530 ± 183	707 ± 95	0.183
eGFR (ml/min/1.73 m²)	188	26 ± 20	23 ± 18	0.296

Data are expressed as mean ± standard deviation (SD) or relative frequency (%). BMI, body mass index; WC, waist circumference; SBP, systolic blood pressure; DBP, diastolic blood pressure; PP, pulse pressure; Hb, haemoglobin; TC, total cholesterol; LDL-C, low-density lipoprotein cholesterol; HDL-C, high-density lipoprotein cholesterol; TG, triglycerides; eGFR, estimated glomerular filtration rate.

Multivariate determinants of CKD with a special emphasis on SCT are presented on Table 5. SCT did not emerge as an independent determinant of CKD; the main determinants were hypertension, diabetes, the MetS and anaemia. The presence of diabetes, hypertension, the MetS and anaemia conferred 2.34-fold (OR: 2.36 95% CI: 1.150–4.454; *p* = 0.001), 2.16-fold (OR: 2.16 95% CI: 1.202–3.892; *p* = 0.001), 1.69-fold (OR: 1.69 95% CI: 1.003–2.965; *p* = 0.038) and 3.12-fold (OR: 2.34 95% CI: 1.202–3.892; *p* = 0.001) greater risk for CKD, respectively, in comparison with patients without these risk factors.

**Table 5:** Multivariate independent determinants of chronic kidney disease

*Variable*	*B*	*SE*	*OR (95% CI)*	*p-value*
Constant	0.227	0.397	–	–
HbAS vs HbAA	0.953	0.810	0.38 (0.559–1.839)	0.235
DM+ vs DM–	1.343	0.282	2.36 (1.150–4.454)	0.001
HT vs NT	0.771	0.300	2.16 (1.202–3.892)	0.001
MetS+ vs MetS–	0.559	0.269	1.69 (1.033–2.965)	0.04
Hb ≥ 12 vs 12 g/dl	–1.015	0.278	0.36 (0.220–0.625)	0.001

B, regression coefficient; SE, standard error; OR, odds ratio; Hb, haemoglobin; HbAS, haemoglobin with sickle cell trait; HbAA, normal haemoglobin; DM, diabetes mellitus; HT, hypertension; NT, normotension; MetS, the metabolic syndrome.

## Discussion

The aim of this cross-sectional study was to assess determinants of CKD with a special emphasis on SCT. Traditional risk factors in isolation or combined as the MetS emerged as the main determinants of CKD; however, SCT was not associated with CKD.

Our finding of increased risk for CKD in the presence of the MetS agrees with the results of previous reports on the determinants of CKD. Cheng *et al.*^27^ found a greater risk of CKD (OR: 1.77 95% CI: 1.18–2.46) in patients with the MetS in comparison with those without this risk factor. A similar increased risk of CKD (OR: 1.55 95% CI: 1.34–1.80) was reported by Thomas *et al.*^28^ and Tanner *et al.*,^29^ respectively. Thomas *et al.*^28^ indicated that the risk of CKD increased with the number of individual MetS components. A higher increased risk of CKD (OR: 2.60 95% CI: 1.68–4.08) in the presence of the MetS was reported by Chen *et al.*^30^ This increased risk of CKD is thought to rely on MetS-associated insulin resistance and subsequent oxidative stress and endothelial dysfunction.^27^,^31^,^32^

SCT was not associated with CKD in the present study. Conflicting reports exist as to whether SCT is a risk factor for the development and progression of CKD.^3^,^5^,^6^-^8^ Earlier small-scale reports suggested SCT to be an independent risk factor for CKD and ESRD.^7^,^8^ Derebail *et al.*^8^ observed among 188 ESRD African Americans on dialysis a greater prevalence of SCT (15 vs 7%, *p* = 0.001) in comparison with that inferred from the newborn haemoglobinopathy screening programme; they suggested SCT to be an independent risk factor for CKD.^5^

Ajayi *et al.*^10^ found in black Africans a greater prevalence of microalbuminuria and proteinuria in type 2 diabetes patients with SCT in comparison with those with normal haemoglobin levels. All these authors speculated that the increased prevalence of SCT could be due to accelerated progression of kidney disease either as a direct consequence of SCT or by HbAS enhancing the deleterious effects of another co-morbid condition, such as diabetes, hypertension or autosomal polycystic kidney disease (APKD).^3^,^5^,^7^,^8^

With reference to methodological issues inherent in these cross-sectional studies and the geographical variations in the prevalence of HbAS, additional examination of SCT has been suggested in well-characterised, geographically diverse populations with advanced kidney disease.5 It may also be interesting to examine the interaction of SCT with other recently identified genetic risks for ESRD in black individuals, such as apolipoprotein 1 (APOL1) and non-muscle myosin heavy-chain 9 (MYH9).^5^

In line with the above suggestions, recent studies such as the present study reported no association between SCT and CKD.^7^ Bleyer *et al.*^26^ found in 376 African American diabetics that those with and without SCT had similar eGFR and prevalence of microalbuminuria. Using multivariate analysis, they noted no difference in the combined outcomes of peripheral vascular resistance, retinopathy and renal failure.

In order to determine whether the HbAS genotype is associated with commonly reported aetiologies of ESRD, Hicks *et al.*^6^ evaluated cases (*n* = 3.258) with ESRD attributed to type 2 diabetes and non-diabetes causes, predominantly hypertension attributed and glomerular disease associated. In addition, relationships between APOL1 G1/G2 nephropathy risk variants and non-muscle MYH9 risk variants (E1 risk haplotype) and SCT were assessed to determine whether interactions between these genes were present. The SCT genotype frequencies were similar in the cases (8.7% in non-diabetic and 7.1% in type 2 diabetes ESRD) and the controls (7.2%). No evidence of association between HbAS and either diabetic or non-diabetic aetiologies of ESRD was detected in this large sample of African Americans. In addition, no evidence of APOL1 or MYH9 interaction with SCT was observed.

The authors suggested both APOL1 and HbS to be associated with susceptibility to nephropathy in autosomal, recessive patterns, with no evidence of risk for nephropathy in individuals heterozygous for risk variants (e.g. those with SCT).^6^ They concluded that African Americans who have a single copy of the HbS gene are not at increased risk for developing non-diabetic or diabetic ESRD or subclinical nephropathy, relative to unaffected individuals.^6^ In addition, nephropathy risk variants in APOL1 function independently from HbS when contributing to non-diabetic ESRD.^6^ In contrast to earlier, smallscale reports using high-performance liquid chromatography (HPLC) to determine HbS, the strengths of this study include the large sample size and direct genotyping for HbS.^6^

The interpretation of the results of our study is confounded by some limitations. The cross-sectional design of the study precludes any causal relationship between CKD and associated risk factors. Moreover, the small sample size did not allow sufficient power to detect any additional associations. Definition of reduced kidney function and CKD was based on a unique determination of serum creatinine. As in earlier smaller studies, HbS determination was based on HPLC instead of direct genotyping of HbS. One wonders to what extent the conclusions of this clinic-based study could be extrapolated to the general population, given the bias in the referral of patients. The findings of our study, however, give some indications about the relationship between SCT and CKD, highlighting the need for a well-characterised study with a large sample of CKD patients.

## Conclusion

In the present case series of black Africans, SCT did not emerge as an independent determinant of CKD. Classic CKD risk factors in isolation or combined as the MetS emerged as the main determinants of CKD.
